# Nontuberculous mycobacterial pulmonary disease presenting as bronchiolitis pattern on CT without cavity or bronchiectasis

**DOI:** 10.1186/s12890-024-03223-2

**Published:** 2024-09-02

**Authors:** Sung Hyun Yoon, Hyung-Jun Kim, Jihang Kim, Junghoon Kim, Jae Ho Lee

**Affiliations:** 1 Department of Radiology, Seoul National University Bundang Hospital, Seongnam, Republic of Korea; 2 Division of Pulmonary and Critical Care Medicine, Department of Internal Medicine, Seoul National University Bundang Hospital, Seongnam, Republic of Korea; 3 Department of Internal Medicine, Seoul National University College of Medicine, Seoul, Republic of Korea

**Keywords:** Bronchiectasis, Bronchiolitis, Nontuberculous mycobacterial infection, Pulmonary infection

## Abstract

**Background:**

This study aimed to investigate the radiological changes in patients with nontuberculous mycobacterial pulmonary disease (NTM-PD) having bronchiolitis patterns on computed tomography (CT).

**Methods:**

We retrospectively reviewed the final diagnosis and radiologic changes of patients suspected of having NTM-PD without cavity or bronchiectasis on CT image, between January 1, 2005 and March 31, 2021. NTM-PD was diagnosed based on the American Thoracic Society and Infectious Diseases Society of America criteria. The initial and final CT findings (bronchiectasis, cellular bronchiolitis, cavity formation, nodules, and consolidation) were compared between patients diagnosed with and without NTM-PD.

**Results:**

This study included 96 patients and 515 CT images. The median CT follow-up duration was 1510.5 (interquartile range: 862.2–3005) days. NTM-PD was recognized in 43 patients. The clinical variables were not significantly different between patients with and without NTM-PD, except for underlying chronic airway disease (*P* < 0.001). Nodule and consolidation were more frequently observed on the initial CT scans of patients with NTM-PD compared with those without (*P* < 0.05). On the final follow-up CT scan, bronchiectasis (*P* < 0.001), cavity (*P* < 0.05), nodule (*P* < 0.05), and consolidation (*P* < 0.05) were more frequently observed in patients with NTM-PD. Among the 43 patients with NTM-PD, 30 showed a radiological progression on CT, with bronchiectasis (*n* = 22) being the most common finding. The incidence of bronchiectasis increased over time.

**Conclusion:**

The bronchiolitis pattern on CT images of patients with NTM-PD showed frequent radiological progression during the follow-up period.

## Background

Nontuberculous mycobacteria (NTM) are ubiquitous organisms found in water, soil, and soil dust [1]. NTM can cause infections of the lungs, sinuses, lymph nodes, and central nervous system in immunocompetent patients, and disseminated disease in patients with innate or acquired immunodeficiency [2, 3]. Among the many organs in which NTM can cause infection, the lungs are the most commonly affected [4]. Recent studies have shown that the incidence and prevalence of nontuberculous mycobacterial pulmonary disease (NTM-PD) are increasing globally [5–10].

Two radiological patterns of NTM-PD have been well recognized: fibrocavitary (FC) and nodular bronchiectatic (NB) [2, 11, 12]. The FC pattern features cavitary lesions mainly in the upper lobes and usually develops in older males with underlying lung diseases [11]. The NB pattern is characterized by bronchiectasis with multiple nodules and tree-in-bud opacities on computed tomography (CT) scans and occurs predominantly in postmenopausal nonsmoking female patients [11]. Consolidative and infiltrative patterns have also been reported [13, 14]; however, most previous studies have focused on FC and NB patterns.

The radiological appearance of NTM-PD is a combination of several findings, including cavity formation, bronchiectasis, consolidation, nodules varying in size, and bronchiolitis (centrilobular nodules with branching opacities) [15–18]. Among these findings, the bronchiolitis pattern is the most common, and was observed in 98% of patients with NTM-PD in a study [15]. In routine CT interpretation practice, it is difficult to specifically suspect NTM-PD when only a mild bronchiolitis pattern is observed without information on respiratory symptoms or a previous CT image. We theorized that a bronchiolitis pattern on CT may indicate an early finding of NTM-PD and may progress to a severe form of NTM-PD. This study aimed to investigate the clinical characteristics and radiological changes in patients with NTM-PD who exhibited a bronchiolitis pattern on CT.

## Methods

### Study population

We retrospectively examined the electronic medical records of patients referred to the outpatient respiratory clinic of a tertiary hospital between January 1, 2005 and March 31, 2021. We followed a stepwise approach to include patients who were referred under suspicion of NTM-PD and whose CT findings showed bronchiolitis without bronchiectasis or cavity formation.

Among the patients referred to the respiratory clinic, we first searched for those whose CT reports had keywords describing bronchiolitis (e.g., bronchiolitis, centrilobular nodule, branching opacity, tree-in-bud, and micronodule) and mentioned NTM infection as a differential diagnosis. Patients with chest CT reports mentioning a cavity or bronchiectasis, and those diagnosed with diffuse panbronchiolitis, pulmonary tuberculosis, tuberculous pleurisy, or sarcoidosis were excluded. Patients with a CT follow-up of < 1 year were excluded. Immunocompromised patients were excluded because pulmonary infections commonly occur in patients with overlapping radiological findings. Any patient suffering from a primary immunodeficiency or hematological malignancy, being treated with steroids or other immunosuppressive drugs for over 4 weeks, or having a history of solid organ or bone marrow transplantation were regarded as immunocompromised. Furthermore, we excluded patients whose CT findings were not likely to be NTM but other diseases with a similar CT appearance (e.g., diffuse panbronchiolitis, bronchopneumonia, or sarcoidosis) after closely reviewing the CT images and final clinical information. A board-certified chest radiologist reviewed all the CT images. The patients were categorized into those diagnosed and not diagnosed with NTM-PD. The diagnosis of NTM-PD was made based on the criteria proposed by the American Thoracic Society/Infectious Diseases Society of America [19]. This retrospective study was approved by the Institutional Review Board of Seoul National University Bundang Hospital (IRB No. B-2309-851-105), and the requirement for a written informed consent was waived.

### Data collection and assessment

Baseline demographics at the time of the first visit were collected from the electronic medical records, including age, sex, body mass index, comorbidities, smoking history, and symptoms. Microbiological test results (NTM species and acid-fast bacilli [AFB] culture) and chest CT images obtained during the follow-up period were also collected.

AFB cultures from respiratory specimens were obtained using standard methods [20]. The microbiological criteria for NTM-PD were met when NTM was isolated from the sputum ≥ 2 times or from the bronchial washing after the first visit. NTM species and subspecies were identified by polymerase chain reaction restriction fragment length polymorphism analysis of the *rpoB* gene.

### CT image review

Chest CT scans were conducted at our institution using multi-detector CTs, with a slice thickness of 3 mm at 2.5 mm intervals. CT images from external hospitals were considered acceptable if they were reconstructed with a slice thickness of < 3 mm and a slice interval smaller than the slice thickness, employing overlapping reconstruction.

The presence of five NTM-related CT findings on the initial and final CT scans was assessed [15]: bronchiectasis, cellular bronchiolitis, cavity formation, nodules, and consolidation. Bronchiectasis was defined as a condition in which the bronchial lumen diameter was greater than that of the adjacent pulmonary artery, without normal bronchial tapering. Cellular bronchiolitis was defined as the presence of centrilobular nodules (< 10 mm in diameter) and branching opacities (i.e., the tree-in-bud sign) observed on CT scans. Nodules were defined as solid oval or round lesions 10–30 mm in diameter. Consolidation was defined as a polygonal (not oval or round) homogeneous opacity that replaced the airspace and obscured the underlying pulmonary structures.

A previously published CT scoring system [15] was slightly modified and used to assess the radiological progression between the initial and final CT images (Table 1): bronchiectasis (maximum score, 6), cellular bronchiolitis (maximum score, 6), cavity (maximum score, 9), nodules (maximum score, 3), and consolidation (maximum score, 3). Since the aim of our study is to compare long-term changes in CT images, we removed the mucus plugging item from the bronchiectasis section of the original scoring system. This adjustment ensures that our scores more accurately reflect chronic irreversible changes rather than fluctuations in inflammation. Radiological progression was defined as present when the final CT score of any finding was greater than the initial CT score. A scoring system was not used to compare the CT images between the groups because the initial CT scores were generally expected to be small. The distribution of radiological abnormalities was analyzed based on the lobe and multilobe involvements and bilaterality. The lingular division of the left lung was considered the middle lobe.

During the image analysis, the original CT images were downloaded and reviewed without radiology report or clinical information, such as NTM diagnosis. The initial and final CT images of patients were independently assessed using a scoring system. After assessment by a board-certified radiologist (S.H.Y.), the results were compared with the original radiology reports written by board-certified radiologists. The time point of progression was evaluated in the patients with NTM-PD who exhibited radiologic progression.

**Table 1:** Computed tomography (CT) scoring system for the assessment of the extent of radiological abnormalities

CT finding	Score 0	Score 1	Score 2	Score 3
Bronchiectasis (6 points)				
Severity^*^	Absent	Mild	Moderate	Severe
Extent^†^	Absent	1–5	6–9	> 9
Cellular bronchiolitis (6 points)				
Severity^‡^	Absent	Mild	Moderate	Severe
Extent^†^	Absent	1–5	6–9	> 9
Cavity (9 points)				
Diameter	Absent	< 3	3–5	> 5
Wall thickness	Absent	< 1	1–5	> 5
Extent^§^	Absent	1–3	4–5	> 5
Nodules (3 points)^†^	Absent	1–5	6–9	> 9
Consolidation (3 points)^†^	Absent	< 3	3–5	> 5

^*^ Mild = bronchus diameter greater than adjacent vessel diameter; moderate = bronchus diameter 2–3 times the vessel diameter; severe = bronchus diameter > 3 times the vessel diameter

^†^ Data represent the number of segments

^‡^ Mild = identifiable, peripheral lung, 1 cm from pleura; moderate = definite, involvement greater than 1–3 cm from pleura; severe = extensive, extending to central lung

^§^ Data represent the number of cavities

### Statistical analysis

Data are presented as numbers (percentages) for categorical variables and medians (interquartile ranges) for continuous variables. Fisher’s exact test was used to compare the categorical variables. The Wilcoxon rank-sum test was used to compare the continuous variables. *P*-values < 0.05 were considered statistically significant. The statistical calculations were performed using the R 4.1.2 (R Foundation for Statistical Computing, Vienna, Austria) software.

## Results

### Patient characteristics

**Fig. 1:**
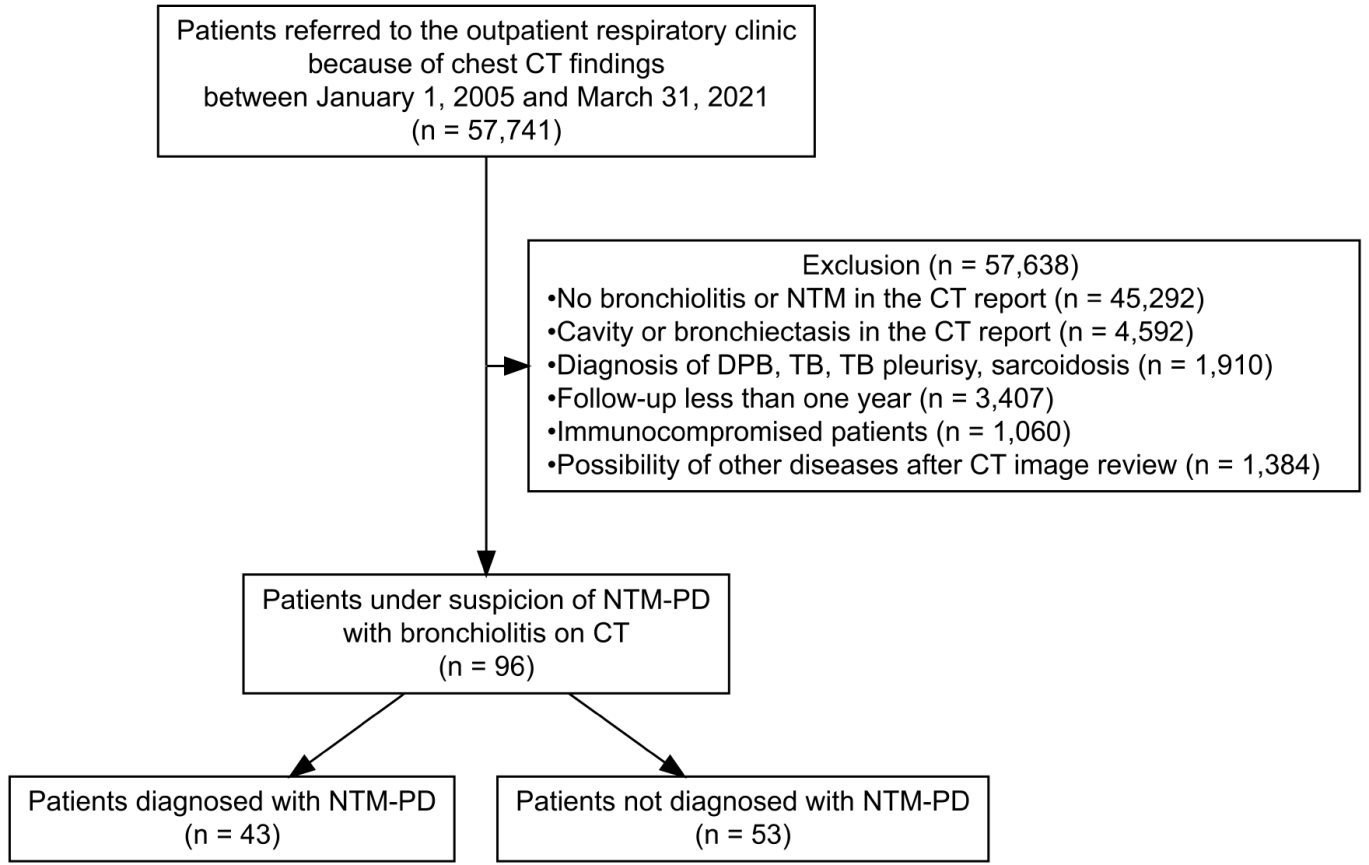
Flowchart of study population. NTM, nontuberculous mycobacteria; TB, tuberculosis; DPB, diffuse panbronchiolitis; CT, computed tomography

Among the 57,734 patients who visited the outpatient respiratory clinic and underwent a chest CT scan during the study period, 96 were suspected to have NTM-PD without bronchiectasis or cavity formation on CT. These patients underwent a total of 515 chest CT scans during the follow-up period.(Fig. 1).

**Fig. 2:**
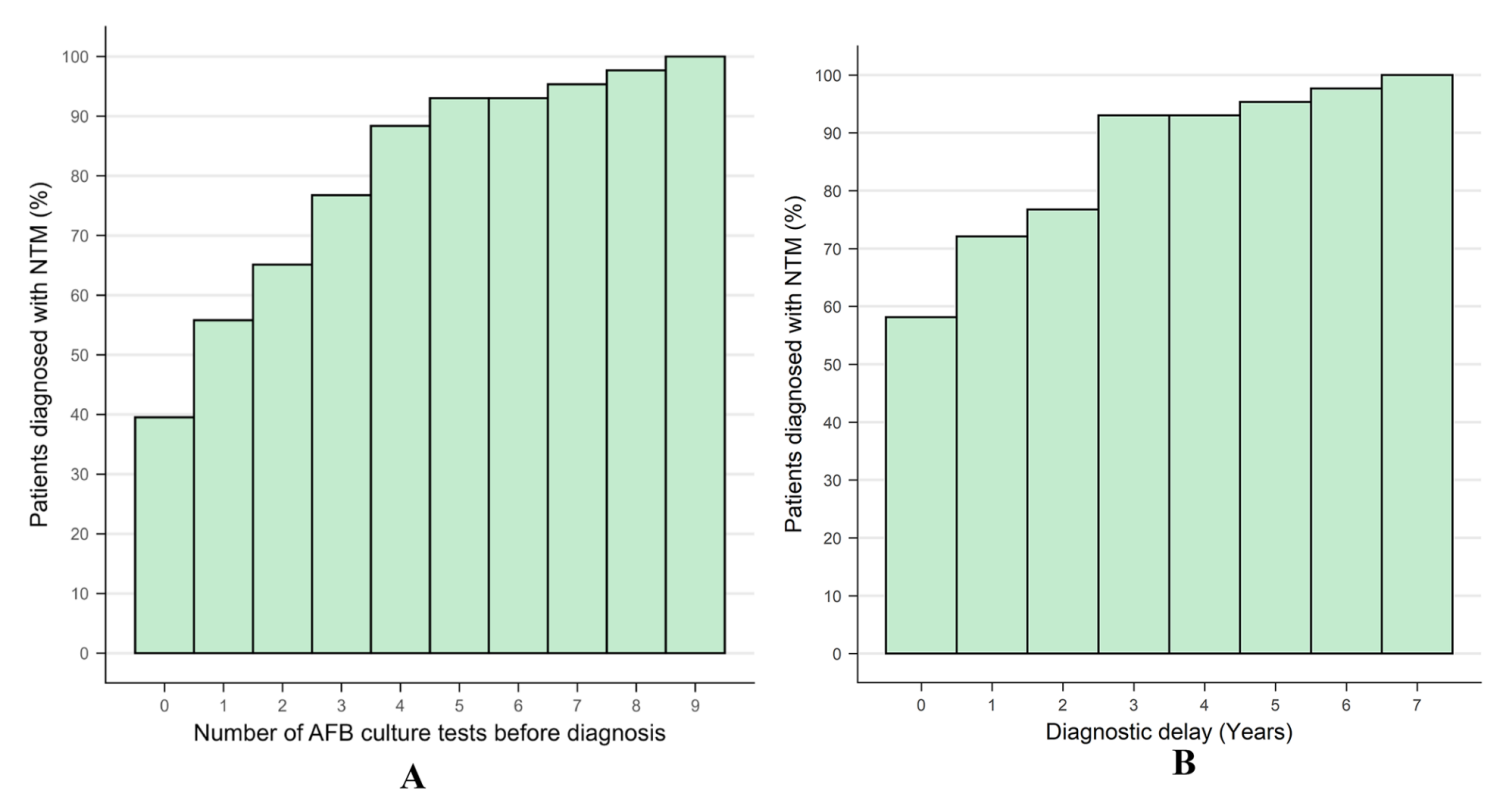
Cumulative diagnosis of NTM in the patients diagnosed with NTM-PD. (**A**) Cumulative diagnosis per the number of negative AFB tests before diagnosis. (**B**) Annual cumulative diagnosis of NTM-PD. NTM, nontuberculous mycobacteria; AFB, acid-fast bacilli

The median CT follow-up duration for 96 patients was 1510.5 days (interquartile range [IQR]: 862.2–3005 days). The median number of CT scans per patient was 4 (IQR: 3–6), and the median CT interval was 221 days (IQR: 61.5–419.5 days). All CT images were considered acceptable for the analysis. During the study period, 43 patients were subsequently diagnosed with NTM-PD and 53 were not, despite repetitive AFB culture tests (Table 2). Among the patients with NTM-PD, 26 patients were diagnosed by having ≥ 2 separate positive sputum culture results, and 17 were diagnosed by having ≥ 1 positive culture result from bronchial washings. None of the patients without NTM-PD met the microbiological criteria for NTM-PD. Among these, 7 patients had only one positive sputum culture result despite undergoing two or more sputum cultures. The remaining 46 patients consistently had negative sputum cultures. Patients with NTM-PD underwent fewer sputum or bronchial washing culture tests before the final diagnosis (*P <* 0.05), although the CT follow-up period was not significantly different. Other clinical variables were not significantly different between the two groups, except for underlying chronic airway diseases (chronic obstructive pulmonary disease (COPD) and asthma) (*P <* 0.001). There was 1 case of COPD and 1 case of asthma in the patients with NTM-PD, and 6 cases of COPD and 12 cases of asthma in the patients without NTM-PD. It took at least five negative AFB culture tests over 3 years until 90% of the NTM patients were diagnosed (Fig. 2).

**Table 2:** Clinical characteristics

	All (*n* = 96)	Patients diagnosed with NTM-PD (*n* = 43)	Patients not diagnosed with NTM-PD (*n* = 53)	*P*-value
Age (years)^*^	63.1 (54.1–70.9)	62.6 (46.8–69.9)	64.5 (57–73.9)	0.112
Sex				0.838
Male	41 (42.7%)	17 (39.5%)	24 (45.3%)	
Female	55 (57.3%)	26 (60.5%)	29 (54.7%)	
BMI (kg/m^2^)^*‡^	21.9 (20.5–24.1)	21.6 (20.4–23.5)	22.2 (20.9–24.7)	0.509
Smoking				0.431
Nonsmoker	63 (65.6%)	30 (69.8%)	33 (62.3%)	
Ex-smoker	26 (27.1%)	11 (25.6%)	15 (28.3%)	
Current smoker	7 (7.3%)	2 (4.7%)	5 (9.4%)	
Chronic airway disease	20 (20.8%)	2 (4.7%)	18 (34.0%)	< 0.001
History of TB	4 (4.2%)	2 (4.7%)	2 (3.8%)	1
Symptom	65 (67.7%)	43 (100%)	22 (41.5%)	< 0.001
CT follow-up (days)^*^	1510.5 (862.2–3005)	1593 (925–3244)	1446 (860–2650)	0.512
Diagnostic delay (days)^*^	117 (15.5–1045.8)	113 (17.5–815)	212 (12–1312)	0.459
AFB test before diagnosis				
Sputum culture^†^	2 (0–18)	1 (0–8)	3 (1–18)	< 0.001
BW culture^†^	0 (0–3)	0 (0–2)	0 (0–3)	< 0.05

Unless otherwise specified, the data are presented as numbers of patients with percentages in parentheses. Diagnostic delay was defined as the time (days) from the day of the initial CT scan to the final diagnosis (or the last AFB culture test in the patients not diagnosed with NTM-PD). AFB test before diagnosis was defined as the number of AFB tests conducted before the final diagnosis (or the total number of AFB tests for patients not diagnosed with NTM-PD)

NTM, nontuberculous mycobacteria; TB, tuberculosis; BMI, body mass index; AFB, acid-fast bacilli; BW, bronchial washing

^*^ Data are presented as medians, with interquartile ranges in parentheses

^†^ Data are represented as medians, with ranges in parentheses

^‡^ Values were missing (Patients diagnosed with NTM-PD:1, Patients not diagnosed with NTM-PD:2)

*P*-values were calculated using the Wilcoxon rank-sum test for age, BMI, CT follow-up period, diagnostic delay, sputum culture, and bronchoalveolar lavage (BAL) culture, and Fisher’s exact test for the other variables

#### Comparison of the CT findings

There was no disparity between the scoring results of CT images and the original radiology reports. Regarding the initial CT findings, nodules and consolidations were present in different proportions, which were more frequently observed in patients with NTM-PD than in those without NTM-PD (*P <* 0.05) (Table 3). The presence of other findings was not significantly different between the two groups. The distribution of radiological abnormalities was not significantly different, except for the upper lobe involvement, which was more frequently observed in patients with NTM-PD.

The frequency of radiological abnormalities on the final CT image showed a significant difference between the groups in four of the five findings (Table 4). Bronchiectasis (*P <* 0.001), cavities (*P <* 0.05), nodules (*P <* 0.05), and consolidations (*P <* 0.05) were more frequently observed in patients with NTM-PD. The distribution of radiological abnormalities did not differ significantly between the two groups.

**Table 3:** Initial chest CT findings of NTM and non-NTM groups

	Patients diagnosed with NTM-PD (*n* = 43)	Patients not diagnosed with NTM-PD (*n* = 53)	*P* value
Bronchiectasis	0 (0%)	0 (0%)	1
Cavity	0 (0%)	0 (0%)	1
Nodule	6 (14.0%)	1 (1.9%)	0.042
Consolidation	13 (30.2%)	4 (7.5%)	0.006
Bronchiolitis	43 (100%)	53 (100%)	1
Involved lobes			
Bilaterality	30 (69.8%)	42 (79.2%)	0.826
Multilobe	39 (90.7%)	46 (86.8%)	0.749
Upper lobe	42 (97.7%)	43 (81.1%)	0.020
Middle lobe^*^	32 (74.4%)	36 (67.9%)	0.508
Lower lobe	29 (67.4%)	41 (77.4%)	0.357

Data are numbers of patients, with percentages in parentheses

^*^ The right middle lobe and lingular division of the left lung were regarded as middle lobes

*P* values were calculated by using the Fisher’s exact test

CT, computed tomography; NTM, nontuberculous mycobacteria

**Table 4:** Last chest CT findings of NTM and non-NTM groups

	Patients diagnosed with NTM-PD (*n* = 43)	Patients not diagnosed with NTM-PD (*n* = 53)	*P* value
Bronchiectasis	22 (51.2%)	7 (13.2%)	< 0.001
Cavity	6 (14.0%)	0 (0.0%)	0.007
Nodule	11 (25.6%)	4 (7.5%)	0.023
Consolidation	16 (37.2%)	7 (13.2%)	0.008
Bronchiolitis	41 (95.3%)	49 (92.5%)	0.688
Involved lobes			
Bilaterality	36 (83.7%)	40 (75.5%)	0.070
Multilobe	36 (83.7%)	44 (83.0%)	1
Upper lobe	41 (95.3%)	45 (84.9%)	0.177
Middle lobe^*^	35 (81.4%)	35 (66.0%)	0.110
Lower lobe	28 (65.1%)	40 (75.5%)	0.367

Data are numbers of patients, with percentages in parentheses

^*^ The right middle lobe and lingular division of the left lung were regarded as middle lobes

*P* values were calculated by using the Fisher’s exact test

CT, computed tomography; NTM, nontuberculous mycobacteria

**Fig. 3:**
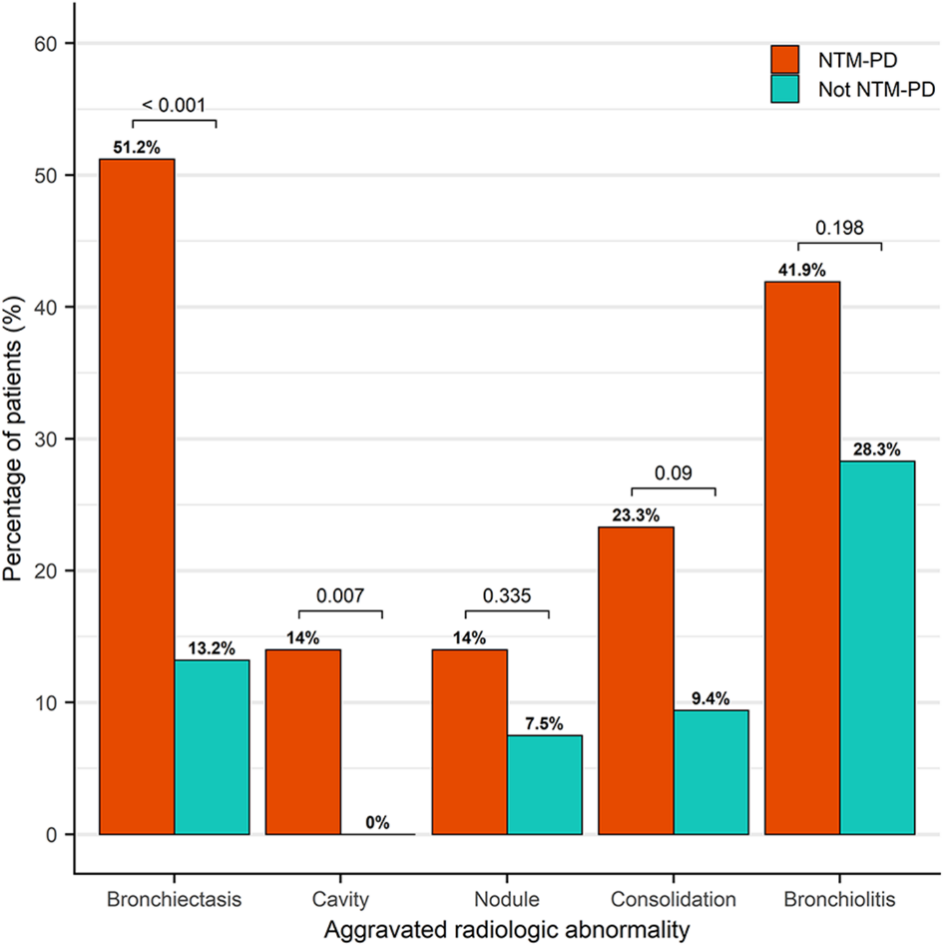
Percentage of patients with aggravated radiological abnormality

**Fig. 4:**
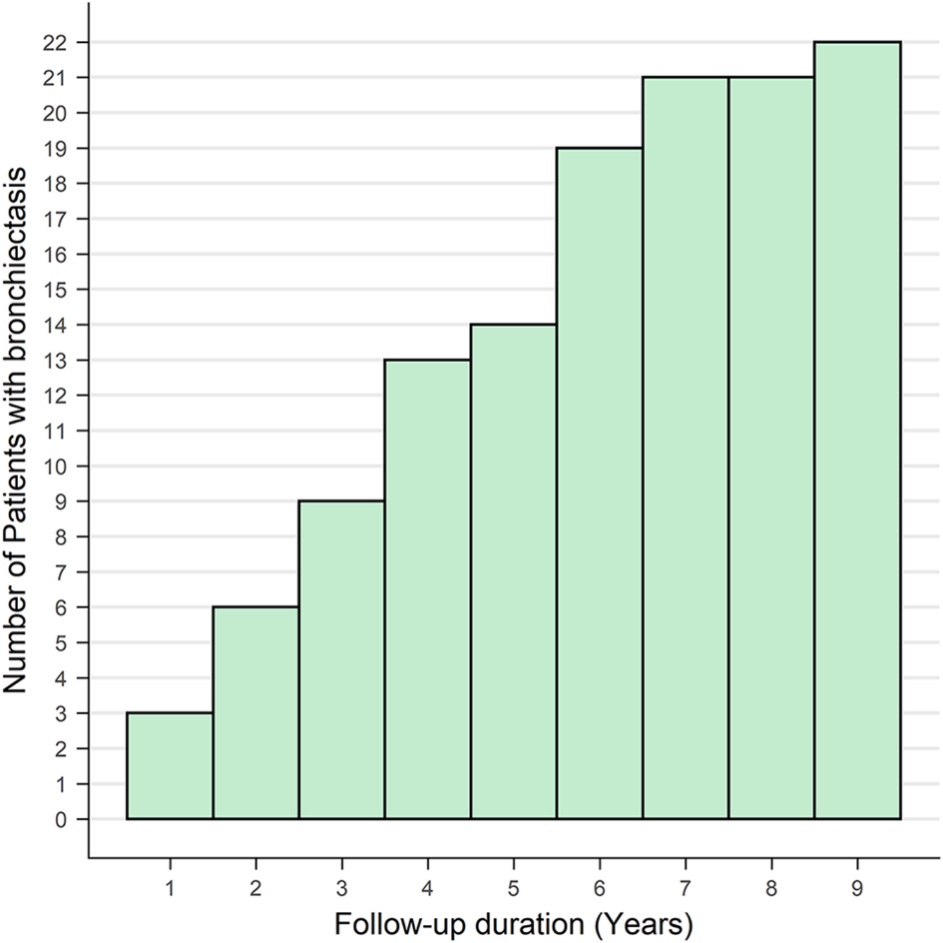
Cumulative incidence of bronchiectasis in patients with NTM-PD. NTM-PD, nontuberculous mycobacterial pulmonary disease

**Fig. 5:**
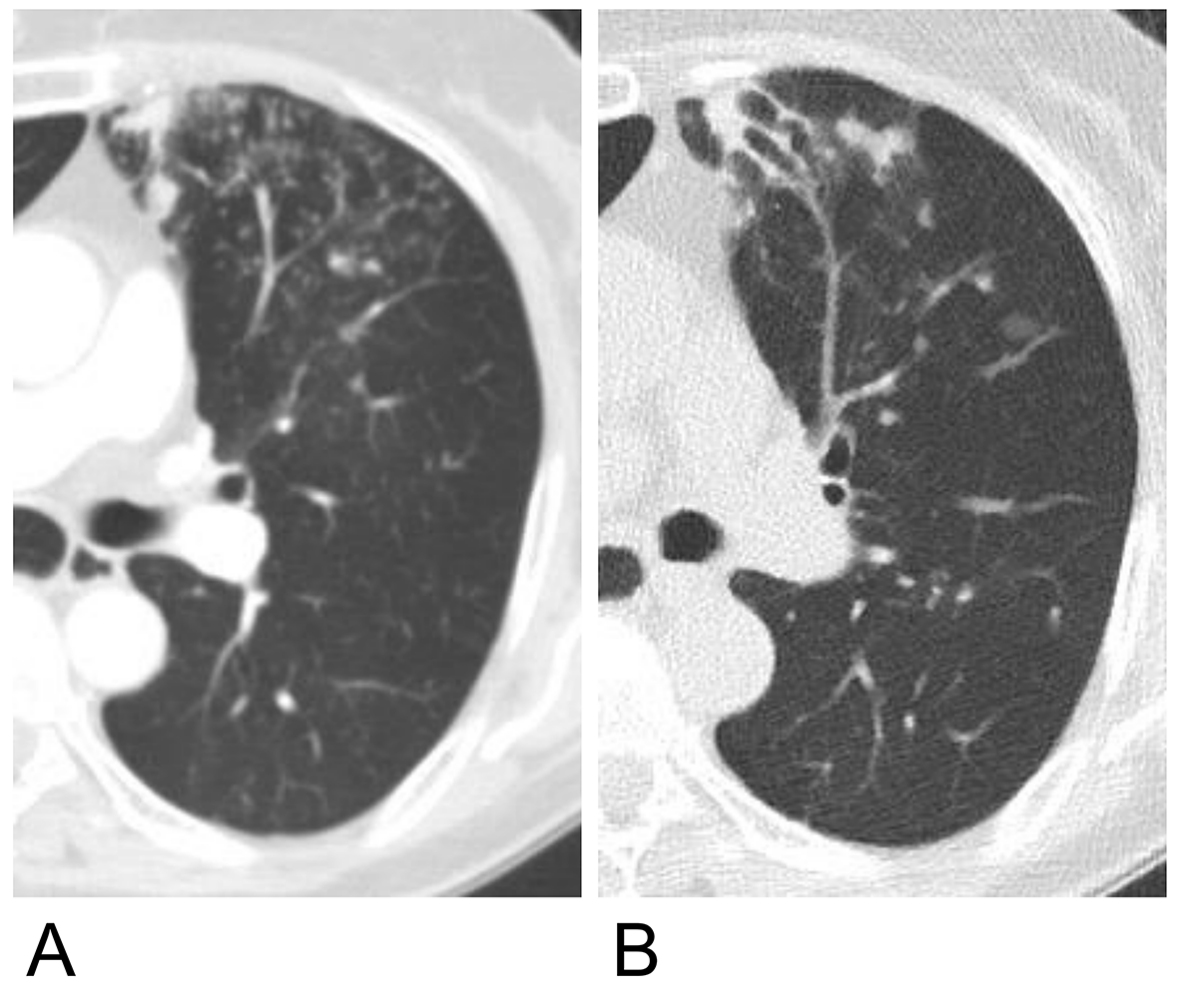
Chest CT images of a female patient. (**A**) The initial CT image showed no bronchiectasis. Centrilobular and small nodules were observed in the anterior segment of the left upper lobe. (**B**) Bronchiectasis became evident after 5 years and centrilobular nodules were still observed but to a lesser extent. CT, computed tomography

**Fig. 6:**
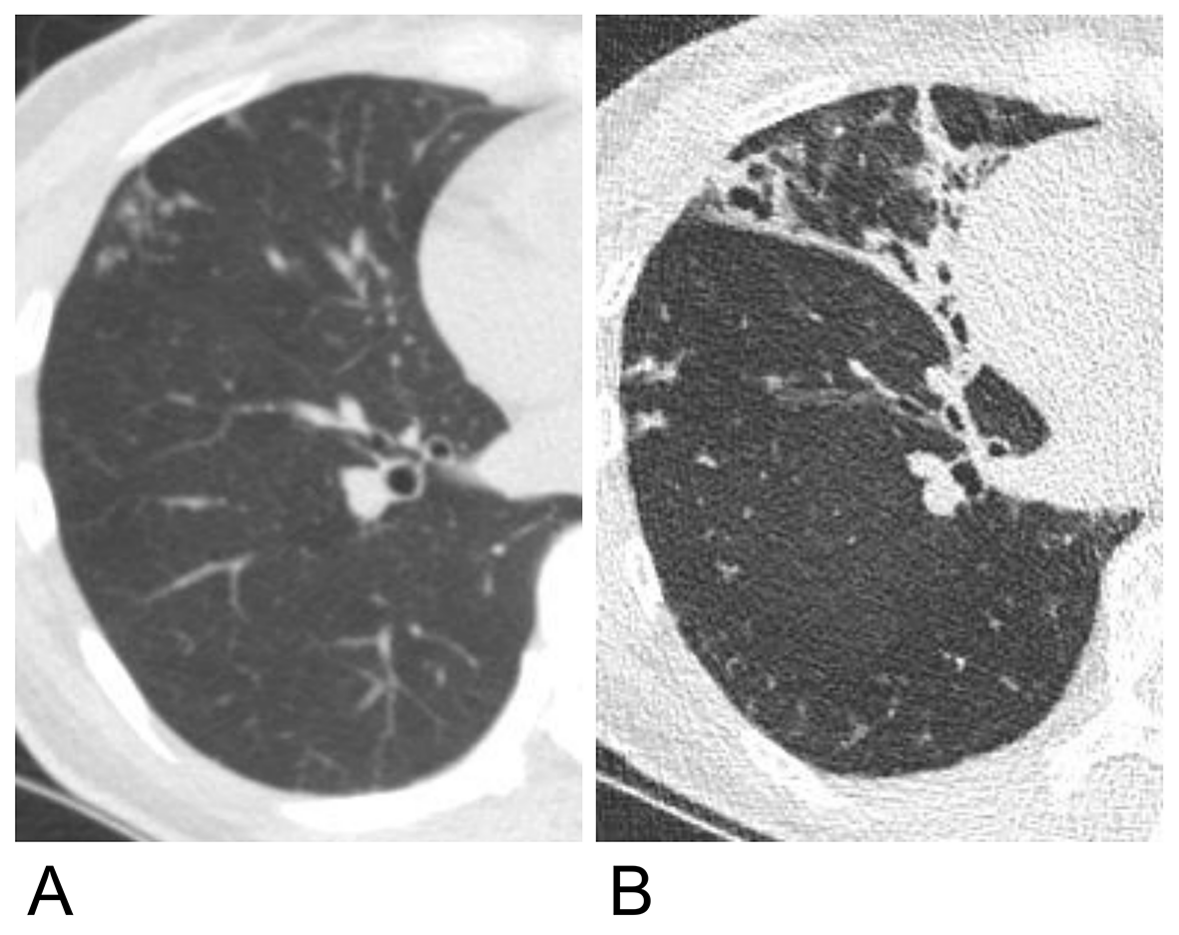
Chest CT images of a female patient. (**A**) The initial CT image showed no bronchiectasis. Centrilobular and small nodules were observed in periphery of the right middle lobe. (**B**) Bronchiectasis became evident after 10 years and centrilobular nodules were still observed. CT, computed tomography

The progression of each finding during the follow-up period was assessed using a scoring system and compared between the patients with and without NTM-PD (Fig. 3). Among the 43 patients with NTM-PD, the CT images of 30 patients (69.8%) showed a radiological progression. In comparison, 22 out of the 53 patients without NTM-PD (41.5%) exhibited radiological progression on their CT images. Additionally, of the 43 patients with NTM-PD, 11 (25.6%) received treatment, with 9 of these patients being in the radiologically progressive group. The mean increase in the total score for radiological abnormalities during the follow-up period was 2.05 in patients with NTM-PD and 0.08 in those without. Only the development of bronchiectasis and cavities differed significantly between the two groups (*P <* 0.01), with a higher frequency in patients with NTM-PD. Notably, the development of bronchiectasis was observed in 51.2% (22/43) of patients with NTM-PD and 73.3% (22/30) of those with radiological progression. The incidence of bronchiectasis increased over time in the NTM group (Fig. 4). The development and progression of bronchiectasis during the follow-up are shown in Figs. 5 and 6. Although the presence of nodules and consolidations was more common in patients with NTM-PD on both the initial and final CT scans, the progression of these two findings was not significantly different between patients with and without NTM-PD.

#### NTM species

The identification of NTM species was performed in 42 of the 43 patients (Table 5). *M. avium* (*n* = 18) was the most frequently encountered species, followed by *M. intracellulare* (*n* = 12) and *M. abscessus* (*n* = 7). The other three patients were infected with *M. avium* complex (*n* = 1), *M. fortuitum* (*n* = 1), and an unidentified NTM (*n* = 1). The radiological progression was observed in more than half of the patients with *M. avium*, *M. intracellulare*, and *M. abscessus* infections. Nearly half of the patients with *M. avium*, *M. intracellulare*, and *M. abscessus* infections developed bronchiectasis during the follow-up.

**Table 5:** Isolated NTM species

	*n*	Radiologic Progression	Bronchiectasis
*Mycobacterium avium*	18	10 (55.6%)	8 (44.4%)
*Mycobacterium intracellulare*	12	9 (75%)	7 (58.3%)
*Mycobacterium abscessus*	7	5 (71.4%)	3 (42.9%)
*Mycobacterium avium* complex (MAC) ^*^	1	1 (100%)	0 (0%)
*Mycobacterium fortuitum*	1	1 (100%)	0 (0%)
Unidentified NTM	1	1 (100%)	1 (100%)
Mixed infection^†^	2	2 (100%)	2 (100%)
Total	42	29	22

Unless otherwise specified, data are numbers of patients, with percentages in parentheses

^*^ Species identification was not possible for *Mycobacterium avium* complex

NTM, nontuberculous mycobacteria

## Discussion

In this study, 96 patients who presented with bronchiolitis on CT scans but without bronchiectasis or cavities were followed up for a median follow-up period of 1510.5 days. Among 43 patients who were confirmed to have NTM-PD, 30 exhibited a radiological progression, mostly due to bronchiectasis. The progression to bronchiectasis and cavities was significantly more common in patients with NTM-PD than in those without.

Although NTM-PD manifest in various forms on CT scans [18, 21], previous studies have primarily focused on two well-known patterns: FC and NB [2, 11, 12]. Consequently, other patterns have received relatively little attention [22]. Based on our clinical experience, we have observed a significant number of patients with NTM-PD who exhibit bronchiolitis without bronchiectasis or cavity on CT. However, only one study specifically investigated the bronchiolitis subtype of pulmonary NTM infections [22]. Additionally, there is a lack of research on the radiological changes that occur from the early to late stages of NTM-PD. Therefore, the early manifestations of NTM-PD have not been thoroughly investigated.

Our study demonstrated that among patients with NTM-PD having a bronchiolitis pattern, the radiological abnormalities progressed over time. Notably, bronchiectasis was the most frequent new finding in patients with NTM-PD with a radiological progression, suggesting that the bronchiolitis pattern may serve as an early indicator of NTM-PD. However, distinguishing between patients with and without NTM-PD using CT scans, when they present solely with bronchiolitis, remains challenging. The only discernible difference based on initial chest CT findings was the slightly higher prevalence of nodules and consolidations (14.0% vs. 1.9% and 30.2% vs. 7.5%, respectively; *P* < 0.05) in patients with NTM-PD. These findings may assist in identifying patients with suspected NTM-PD who require a meticulous observation with additional follow-up CT scans.

Although the correlation between bronchiectasis and NTM-PD is well-established, the exact cause remains uncertain. In a study involving 221 patients with non-NTM bronchiectasis, 31 were diagnosed with NTM-PD during a median observation period of 37 months [23]. Moreover, individuals with bronchiectasis are reportedly 50–75 times more likely to have NTM infections compared to those without [24]. In contrast, pulmonary NTM infections may lead to bronchiectasis. According to the U.S. Bronchiectasis Research Registry, approximately 60% of patients with bronchiectasis had a history of NTM-PD or NTM cultured from sputum samples, and patients with NTM were more likely to exhibit diffusely dilated airways [25]. Another study that focused on patients with bronchiectasis revealed that patients with NTM-PD had a higher number of pulmonary segments affected by bronchiectasis [26]. No study has yet directly shown bronchiectasis development in NTM-PD via serial CT scans. Our study closely reviewed the CT images of patients with NTM-PD who initially presented with bronchiolitis and observed the development of bronchiectasis. The incidence of bronchiectasis increased over time in patients with NTM-PD, strongly indicating pulmonary NTM infection as a direct cause of bronchiectasis.

Diagnosis and surveillance of patients with NTM-PD can be challenging due to nonspecific clinical signs and the lack of a pathognomonic test [11, 27]. If NTM-PD is highly suspected and the initial tests are nondiagnostic, additional sputum cultures should be obtained. However, repeated cultures often show negative results. In addition, no consensus has been reached regarding the number of additional cultures and the length of intervals between the cultures to exclude NTM-PD safely. Considering that it took a minimum of five AFB tests over 3 years to diagnose 90% of patients with NTM-PD (Fig. 2), we recommend considering at least five repetitive AFB culture tests within 3 years for patients exhibiting a bronchiolitis pattern and suspected NTM-PD.

This study had several limitations. First, as the study was conducted at a respiratory clinic in a single tertiary hospital, our results may not be generalizable to a broader population. Second, this study was retrospective and involved a relatively small sample size. We included patients based on CT reports indicating bronchiolitis and mentioning NTM. Some patients without explicit mentions of NTM in the initial CT report might have been excluded, though this is likely rare. As patients were referred to the respiratory clinic and NTM is prevalent in South Korea, diagnosing NTM is of significant interest to both clinicians and radiologists. Consequently, mentions of NTM often emerged in subsequent follow-up CT reports, and these cases were included in this study. Further research using larger cohorts and a prospective approach is necessary to validate and expand upon our observations. Third, the definitive diagnosis of patients without NTM-PD remains unclear. With repeated AFB testing and longer follow-up durations, it is possible that some individuals initially not diagnosed with NTM-PD may later be diagnosed with NTM-PD or other related conditions.

## Conclusions

The CT images displaying bronchiolitis patterns in patients with NTM-PD exhibited frequent radiological progression during the follow-up period. The most common observation among patients with NTM-PD showing radiological progression was the development of bronchiectasis. These results strongly indicate that pulmonary NTM infection directly leads to bronchiectasis. NTM-PD manifesting as a bronchiolitis pattern on CT scans necessitates thorough follow-up, including additional CT scans.

## Data Availability

The datasets used and/or analysed during the current study are available from the corresponding author on reasonable request.

## Abbreviations

FC: Fibrocavitary
NB: Nodular bronchiectatic
NTM-PD: Nontuberculous mycobacteria pulmonary disease
NTM: Nontuberculous mycobacteria
CT: Computed tomography
AFB: Acid-fast bacilli
IQR: Interquartile range
